# A person-centred care transition support for people with stroke/TIA: A study protocol for effect and process evaluation using a non-randomised controlled design

**DOI:** 10.1371/journal.pone.0299800

**Published:** 2024-03-14

**Authors:** Sebastian Lindblom, Maria Flink, Lena von Koch, Malin Tistad, Una Stenberg, Marie Elf, Axel C. Carlsson, Ann Charlotte Laska, Charlotte Ytterberg

**Affiliations:** 1 Department of Neurobiology, Care Sciences and Society, Karolinska Institutet, Stockholm, Sweden; 2 Theme of Women’s Health and Allied Health Professionals, Karolinska University Hospital, Stockholm, Sweden; 3 Theme of Heart & Vascular and Neuro, Karolinska University Hospital, Stockholm, Sweden; 4 School of Health and Welfare, Dalarna University, Falun, Sweden; 5 The Norwegian National Advisory Unit on Learning and Mastery in Health, Oslo University Hospital, Oslo, Norway; 6 Frambu Centre for Rare Disorders, Siggerud, Norway; 7 Academic Primary Health Care Centre, Region Stockholm, Stockholm, Sweden; 8 Department of Clinical Sciences Danderyd Hospital, Karolinska Institutet, Stockholm, Sweden; University of Pretoria, SOUTH AFRICA

## Abstract

**Introduction:**

Care transitions following a stroke call for integrated care approaches to reduce death and disability. The proposed research described in this study protocol aims to evaluate the effectiveness of a person-centred multicomponent care transition support and the process in terms of contextual moderators, implementation aspects and mechanisms of impact.

**Methods:**

A non-randomized controlled trial design will be used. The intervention includes person-centred dialogue intended to permeate all patient-provider communication, various pedagogical modes of information, a person-centred care and rehabilitation plan, and a bridging e-meeting to prepare patients for homecoming. Patients with stroke or TIA who are to be discharged from the participating hospitals to home and referred to a neurorehabilitation team for continued rehabilitation will be included. Follow-ups will be conducted at one week, 3 months and 12 months. Data will be collected on the primary outcome of perceived quality of the care transition, and on the secondary outcomes of health literacy, medication adherence, and perceived person-centeredness. Data for process evaluation will be collected through semi-structured interviews, focus groups, participatory observations, and the Normalisation Measure Development Questionnaire.

**Discussion:**

The study will provide insights on implementation, mechanisms of impact, contextual moderators, and effectiveness of a care transition support, targeting a poorly functioning part of the care trajectory for people with stroke and TIA.

**Clinical trial registration:**

ClinicalTrials.gov Identifier: NCT05646589.

## Introduction

Stroke is a life-threatening condition that needs acute treatment [1]. Strong evidence has shown that stroke care should initially be supplied at hospital stroke units [2]. Sweden is considered to have efficient medical acute stroke care [3], with short hospitalizations and follow-up in primary or secondary care. Thus, the stroke chain of care always implies a transition from inpatient care to outpatient care organized by primary or secondary care, i.e., a shift in responsibility from one healthcare setting to another.

For the patients and their significant others, an uncoordinated care transition may be a burden, especially when they lack experience in navigating the healthcare system [1]. Cognitive impairments, post-stroke fatigue and depression [4], and the sudden onset of stroke or transient ischemic attack (TIA), often render patients and their significant others unprepared for the care transition to their home [5, 6]. In addition, the short hospital stay leaves little time to participate in transition planning [7, 8], leading to a sense of being abandoned to a new and complex life situation after discharge [7]. The Swedish Health and Medical Service Act states that care providers are responsible for coordinating care. Nevertheless, a great responsibility falls on the patient and significant others to coordinate the care transition [9] and few arrangements support the ability of the patients to participate in their care or self-management after discharge [10, 11].

Studies have shown that there are gaps between the information provided by healthcare professionals and the information that patients can understand and apply. Although 90% of recently discharged older people stated that they had understood the discharge information, 40% could not recall their diagnosis and 23% could not recall their planned follow-up [12]. Another study identified that 62% of recently discharged older persons could not recall their new medications [13]. Our pre-studies have shown that present care transitions are not tailored to support the patient’s understanding of the health information that is needed for self-management after hospital discharge [7, 8]. Self-management of secondary stroke prevention, for example medications, is crucial, as recurrent strokes account for 21% of all strokes [14]. Hence, persons who do not understand information supplied at the time of hospital discharge are at risk of another stroke, since they may not adhere to the prescribed secondary stroke prevention treatment. Consequently, at each patient-provider meeting at the hospital, it is critical to ensure that patients understand the health information supplied, to enable self-management of secondary prevention [15].

As part of Sweden’s national system for knowledge-driven management within healthcare, a national person-centred and cohesive care process for stroke and TIA has been developed. The cohesive care process aims to provide equal and effective care based on the best available knowledge. This emphasises the need to develop tailored communication modes, to meet the various information needs of persons with stroke and TIA [16]. From an equality perspective, it is crucial that all patients, regardless of communicative skills and cognitive capacity, not only receive but also understand and have knowledge and competence to use the health information.

The knowledge and competence that enables people “to access, understand, appraise and use information and services in ways that promote and maintain good health” has been defined by the World Health Organization (WHO) as health literacy [17]. WHO emphasizes that health literacy does not only depend on the individual’s ability, the capacity of healthcare organizations to provide services that support the patient’s knowledge and competence is equally important [17]. Low health literacy, which is common after stroke [18], is associated with reduced adherence to medical advice, greater healthcare utilization, and among older persons also an overall poorer health status and higher mortality [19]. Thus, it has been strongly suggested that to enhance self-management after stroke, healthcare services should be tailored to meet the varying levels of patient health literacy [20]. To improve patients’ self-management of secondary prevention, a recent systematic review concluded that “future research should focus on the development of more effective interventions, to translate secondary prevention recommendations into practice” [21]. Therefore, this study translates the intention of the Swedish Health and Medical Service Act, the recommendations of the Swedish person-centred and cohesive care process for stroke and TIA, and recommendations for secondary stroke prevention, into an adaptive person-centred multicomponent care transition intervention, co-designed to fit in practice and tailored to patient needs.

### The intervention

**Fig 1:**
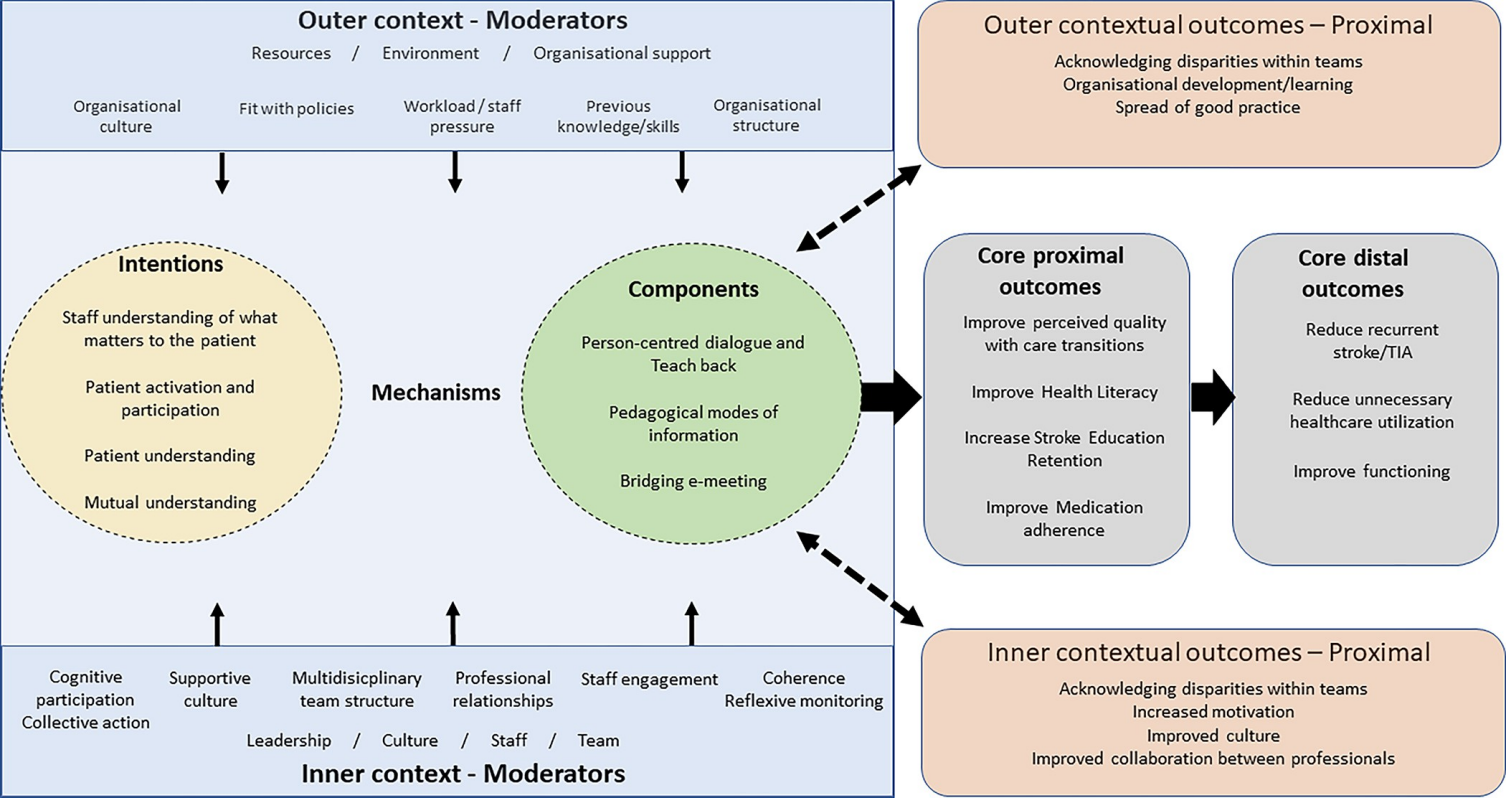
A type 4 logic model, mapping the mechanisms, activities, moderators, and anticipated outcomes of the person-centred multicomponent care transition intervention.

The intervention was developed according to the Medical Research Council framework of complex interventions [22], which calls for phased and iterative approaches in the design and evaluation. Hence, the intervention has been developed by identifying the evidence base for the care transition literature [23–26], understanding local needs and context through our pre-studies [7, 8, 27], and by conducting a co-design process [28] with patients, significant others, and healthcare professionals. The intervention has been tested in a feasibility study investigating fidelity, acceptability, and implementation aspects [29, 30]. Preliminary results show that the intervention is feasible from the perspective of healthcare professionals and patients, and that the proposed methods for evaluation are suitable for patients who recently had a stroke, as well as for their significant others [30]. The intervention has been developed considering both the patient perspective and that the intervention should be context-sensitive and feasible to adapt by the healthcare professionals in diverse organizational settings. Based on the work on building program theory for complex interventions that adapt to context by Mills et al., [31], we constructed a type 4 logic model, mapping the mechanisms, activities, moderators, and anticipated outcomes, which is presented in Fig 1 and described in the text below.

The intervention targets how healthcare professionals can improve the quality of the care transition and support health literacy for self-management of secondary prevention for persons who are to be discharged from hospitals after stroke or TIA. The core intention of the intervention therefore aims to create a person-centred care transition where:

- Staff understand what matters to the patient and tailor their efforts to the unique needs, values, preferences, and resources of the individual.
- Patients are active participants and co-creators in decisions about their care and rehabilitation (provided that is what they desire).
- Patients understand their health condition and what is happening to them.
- Patients understand the prescribed secondary prevention treatment and self-management.
- Patients understand what to expect after discharge in terms of follow-up and rehabilitation.
- A mutual understanding between patients and healthcare professionals is created.

### Intervention components

The intervention includes a set of components that will stimulate the fulfilment of the core intentions. The components include a person-centred dialogue intended to permeate all patient-health professional communication during the acute hospital stay and the continued rehabilitation in the home environment. The person-centred dialogue consists of three different parts [32, 33]: 1) The patient part focuses on the patient’s narrative of “What matters to the patient?” This involves the exploration of patient needs, values, preferences, resources and expectations by the healthcare professionals; 2) The healthcare professional part focuses on the provision and assurance of understanding of information based on the needs of the patient. The objective is to support the patient’s understanding of their health condition, including management of secondary prevention; and 3) The mutual part focuses on a summary and shared understanding of both the patient and professional perspective and mutual agreement on a plan forward. Teach Back-methodology [34, 35] is used in all three parts of the person-centred dialogue. The intervention also includes various pedagogical modes of information: informative recorded videos and written information leaflets with a presentation of the neurorehabilitation team; plans for follow-ups and rehabilitation after discharge; a structured discharge letter on secondary preventive medications, the health condition, and plans for follow-ups. The information is intended to be used at the discharge encounter. Further, a bridging e-meeting is offered to prepare patients for homecoming. The bridging e-meeting before discharge includes the patient, their significant other, healthcare professionals at the hospital and the neurorehabilitation teams in primary care.

### Intervention mechanisms and contextual moderators

As intervention mechanisms are "the interaction between program resources (components) and the ways that participants interpret and respond (or not) to them; an explanatory account of how and why programs give rise to outcomes; hidden, but still real shaped by and interconnected with context" [36], we can only hypothesize about potential mechanisms contributing to our intended outcomes. However, based on our pre-studies, co-design process, and feasibility study, we have gained insight about mechanisms as well as outer and inner context moderators that can shape intervention components and contribute to outcomes, illustrated in Fig 1.

Several potential contextual moderators might exert an influence in the outer and inner context, as suggested in Fig 1. These contextual moderators can both have a positive and negative influence on the implementation, program activities and mechanisms. The feasibility testing made it evident that the intervention is context-sensitive, and the embedding of the intervention varied between settings and healthcare professionals, indicating the need for non-linear evaluation methods.

The overall aim is to evaluate the co-designed adaptive multicomponent care transition intervention. We hypothesise that the person-centred multicomponent care transition will improve the patients’ perceived quality with care transitions, and knowledge and competence to use health information, i.e., health literacy, which in turn may reduce recurrent stroke/TIA and healthcare utilization during the first year after stroke, as well as alleviating caregiver burden for significant others.

Research questions (RQ):

1. Does a person-centred multicomponent care transition intervention have an effect on perceived quality with care transitions, health literacy, collected medications, medication adherence, perceived person-centeredness, functioning, recurrent stroke/TIA, healthcare utilization and caregiver burden?
2. What are the experiences of the intervention components and the implementation process?
3. How does the intervention get adapted and implemented in practice?
4. What contextual moderators and mechanisms of the intervention can likely explain the potential effects of the intervention?

## Materials and methods

### Theory

#### Complexity theory

With the complexity of transitional care, we acknowledge that the intervention involves many adaptive aspects that need to be implemented in a dynamic and complex system. The intervention involves communication and interaction between several agents, i.e., patients with various needs, healthcare professionals and also between different professions in multiple settings—all with various knowledge and competences for communication. Such complexity, defined as a “dynamic and constantly emerging set of processes and objects that not only interact with each other, but come to be defined by those interactions” [37] calls for new approaches in relation to implementation and the evaluation of impact. Based on knowledge generated from our feasibility studies, these agents interact dynamically in a non-linear fashion, which contributes to an unpredictability in how the intervention is embedded and delivered in practice. The behaviours and interactions between these agents contribute to the emergence of the system and this is fundamental to how the intervention behaves [38]. As systems are embedded in a wider context of nested systems that relate to and interact with each other, it is of particular importance to consider the influence of the setting and context when implementing and evaluating interventions in healthcare. Further, as agents and systems co-evolve and self-organise to best fit with the context and the environment [39], the intervention is expected to be adapted differently by and between different agents and contexts. We therefore conclude that the complex, dynamic system of care transitions has outgrown the use of reductionist scientific methods. We must therefore apply methods with a complexity lens that best deals with uncertainty, unpredictability, and generative causality [40].

#### Normalization process theory

To be able to comprehend the uncertainty, dynamics and emergency that implementing an adaptable intervention in a complex system involves, we also need to consider the process of implementation. To facilitate implementation and understand how the healthcare professionals operationalize the intervention, we will use Normalization Process Theory (NPT) as a guide [41]. NPT is a theory of implementation that focuses on what people do, i.e., concentrates attention on the individual and collective behaviour of the implementation process. NPT will be used to understand the social organization of the implementation and how the intervention is embedded and sustained in everyday practice [42, 43]. NPT describes four generative mechanisms; coherence, cognitive participation, collective action and reflexive monitoring [41]. Coherence entails sense-making, i.e., actions to promote professionals’ understanding of how the new practice, the person-centred multicomponent intervention, differs from the usual practice. Cognitive participation entails building and sustaining a community of practice around the new intervention. Collective action is what people do to operationalize and embed the intervention in clinical practice. Reflexive monitoring is the appraisal of how the intervention is understood and assessed by the professionals operationalizing it, which involves evaluating the effects, determining the value of the intervention, and modifying the intervention if needed.

### Study design

We will apply a non-randomized controlled trial design, which is well suited for assessing the effectiveness of complex interventions and allows for a detailed process evaluation. Randomization at the patient level is not suitable, due to the risk of contamination. Further, the use of a non-randomized controlled trial design makes it possible to consider the roles of mechanisms, context, and the complexity of transitional care, during implementation and evaluation. We will use a research design with two intervention sites and two control sites, including multiple follow-ups over a period of one year [44] and, in two parallel phases, we will focus on evaluation of both effect and process. The SPIRIT Checklist was utilized to ensure high-quality reporting of this protocol, for detailed information see S1 Appendix.

### Phase 1: Evaluation of effect

Research question:

1. Does a multi-component care transition intervention have an effect on perceived quality of care transitions, health literacy, collected medications, medication adherence, perceived person-centeredness, functioning, recurrent stroke/TIA, healthcare utilization and caregiver burden?

#### Setting

The intervention will be conducted in the Stockholm region at one acute stroke unit and one geriatric stroke ward and will involve the corresponding neurorehabilitation teams in primary care. Another acute stroke unit and geriatric stroke ward, with the corresponding neurorehabilitation teams in primary care, will serve as control sites. Patients at the control sites will receive standard care transitions. Based on our pre-studies, “standard care transitions” means that the care transition is initiated by an electronic referral from allied health professionals at the hospital to the receiving neurorehabilitation team. The referral notifies the neurorehabilitation team about the patient’s discharge. The teams are then obliged to contact the patient within 48 hours after hospital discharge. Further, a discharge meeting is held with the patient and responsible physicians. Patients receive a standard discharge letter and an informational leaflet about the neurorehabilitation team.

#### Participants

*Patients*. We will include patients who have had a first time ever, or recurrent, stroke or TIA who are to be discharged from the participating hospitals to home and referred to a neurorehabilitation team for continued rehabilitation. The patients must be able to give informed consent by themselves. The patients will be informed of the study and invited to participate at the hospitals by a research assistant. The research assistant will provide oral and written information about the study and will obtain informed consent from the patients who agree to participate.

*Significant others*. The included patients will be asked if they are willing to name a significant other for invitation to participate in the study. The significant others will be mailed written information about the study, including an informed consent form and a pre-stamped envelope. Significant others who return a signed consent will be included in the study. Patients will remain included if they do not have or do not want to name a significant other.

#### Data collection

**Fig 2:**
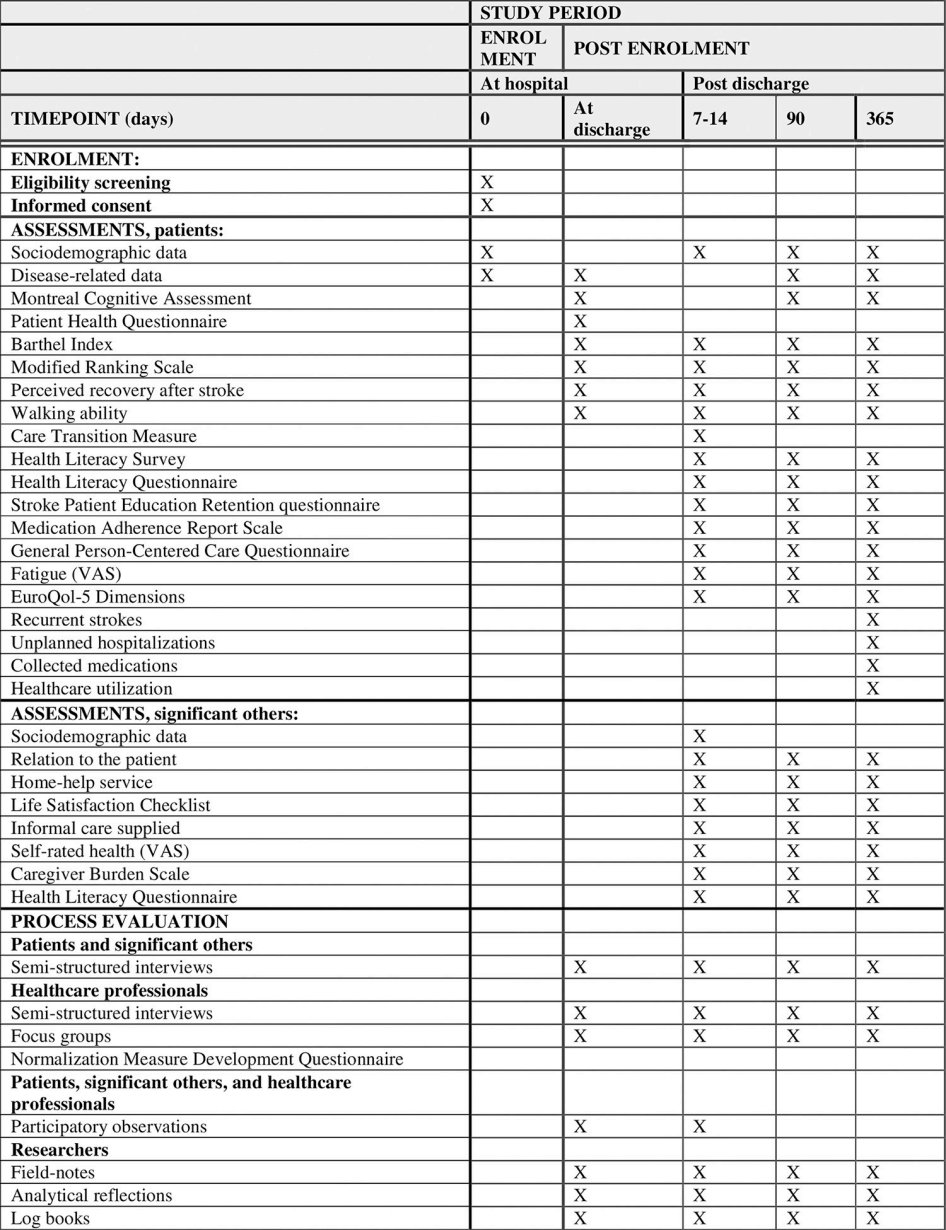
SPIRIT schedule of enrolment, data collection and assessments.

Data will be collected using questionnaires according to a study-specific protocol. Timeline over data collection and information about specific questionnaires can be seen in Fig 2.

*Patient data*. At baseline sociodemographic and disease-related data (e.g., stroke severity, co-morbidities) will be collected from hospital records and questionnaires. Data on patient functioning will be collected through questionnaires; cognitive function will be assessed using the Montreal Cognitive Assessment (MOCA), depression using the Patient Health Questionnaire (PHQ-2), activities of daily living using the Barthel Index (BI), recovery after stroke using a visual analogue scale, level of disability using the modified Ranking Scale (mRS), and walking ability using a single-item question. This baseline data will be collected by the research assistants.

One to two weeks after discharge, data will be collected on the primary outcome of perceived quality of care transitions using the Care Transition Measure (CTM). We will also assess secondary outcomes; health literacy using the Health Literacy Questionnaire (HLQ) and the Health Literacy Survey (HLS), stroke education retention using the Stroke Patient Education Retention questionnaire (SPER), and medication adherence using the Medication Adherence Report Scale (MARS), perceived person-centeredness using the General Person-Centered Care Questionnaire (GPCC-Q). Background and descriptive information will be collected on fatigue using a visual analogue scale, depression using the PHQ-2, level of disability using the mRS; activities of daily living using the BI, and perceived recovery after stroke using a visual analogue scale. We will also ask patients about any new or changed prescribed medications they have received after discharge, as well as perceived met and unmet needs of care and rehabilitation.

At 3 and 12 months the same instruments used at baseline and 1–2 weeks after discharge will be used to collect data on health literacy, stroke education retention, medication adherence, perceived person-centeredness, fatigue, depression, stroke severity, activities of daily living, recovery after stroke, and cognitive function. In addition, data will be collected on health-related quality of life using the EuroQol-5Dimensions (EQ-5D). We will ask patients about any new or changed prescribed medications they have received after discharge, as well as perceived met and unmet needs of care and rehabilitation.

At 12 months, data on number of recurrent strokes, unplanned hospitalizations and collected medications during the first year after stroke will be collected from Region Stockholm’s database (VAL).

*Significant other data*. After written consent, sociodemographic data, relation to the patient, home-help service, life satisfaction using the Life Satisfaction Checklist, information received, informal care supplied, and self-rated health using a visual analogue scale will be collected through a study specific protocol, and data on caregiver burden [45], and health literacy will be gathered using the HLQ. Data will be collected at 1–2 weeks, 3- and 12-months following hospital discharge.

#### Sample size

Results from our pre-studies show that participants had a mean of 62 points in the care transition measure and standard deviation of 21. Based on the estimation that satisfaction will be a mean of 72 in the person-centred multicomponent care transition, we will need to recruit 70 patients (80% power, p = 0.05, 2 sided). Allowing for 20% drop-out, we will need to recruit in total 84 patients per group, 168 patients in total.

#### Analyses

Patients in the intervention group will be compared to the control group using intention-to-treat and per-protocol analysis. Regression models adjusting for covariates (e.g., age, gender, stroke severity, and other disease-related data) will be used for analyses of primary and secondary outcomes. Analyses will be performed and reported in accordance with the Consolidated Standards of Reporting Trials guidelines for non-randomized controlled trials [46].

### Phase 2: Process evaluation

The overall aim of the process evaluation is to explore contextual moderators, implementation aspects, and mechanisms of impact that might explain the potential effects of the multi-component care transition intervention.

Research questions:

2. What are the patients’, significant others’, and healthcare professionals’ experiences of the intervention components and the implementation process?
3. How does the intervention get adapted and implemented in practice?
4. What contextual factors and mechanisms of the intervention can likely explain the potential effects of the intervention?

#### Design

Mixed-method process evaluation.

#### Participants

Qualitative interviews and observation: We will use theoretical sampling [47] to be able to explore the behaviours and interactions between patients, significant others and healthcare professionals in different settings and how the emergence of the system contributes to how the intervention behaves, i.e., is operationalized and adapted in practice.

Quantitative data: All healthcare professionals at the intervention sites will be asked to participate in data collection on implementation aspects and fidelity to the intervention.

#### Data collection

Patients and significant others: Semi-structured interviews with open-ended questions will be held with patients and significant others. Patient and significant other interviews will target their experiences of the care transition, including how they understood information and their perspectives on their interaction with healthcare professionals. For patients at intervention sites, interviews will also target intervention components. To support theoretical sampling and theory building, data will be collected in an iterative, comparative, and interactive way [48].

Healthcare professionals: Semi-structured interviews and focus groups with open-ended questions will be conducted. The healthcare professionals will be asked to describe their experiences of the care transition; and for healthcare professionals in intervention sites, the use of the intervention in everyday clinical practice including the implementation will be explored. Data from healthcare professionals on the process of implementation and maintenance of the intervention will be collected with the Normalization MeAsure Development Questionnaire (NoMAD), which is based on the NPT [49]. NoMAD will be used from the intervention start to iteratively assess and monitor the implementation of the intervention. Data on dose and fidelity to the intervention components will be collected using self-reports from the healthcare professionals.

Patients, significant others, and healthcare professionals: The interaction between patients, significant others, and healthcare professionals will be observed at the hospital and during encounters with the neurorehabilitation team at home. Observations and subsequent interviews will be used to understand how the intervention components are operationalized by healthcare professionals, to capture the interaction between intervention components and the ways that healthcare professionals interpret and respond to them. We will explore the mechanisms of how and why intervention components give rise to outcomes and how contextual moderators influence the implementation, intervention components and mechanisms. Further, interviews on implementation aspects will be guided by NPT and will focus on how people make sense of the intervention (coherence), how people make it work in practice (collective action), how people assess and decide whether it is worth the effort (reflexive monitoring), and how healthcare professionals get involved and stay committed (cognitive participation) [41]. Field-notes and analytical reflections will be made by the responsible researchers.

All interviews and observations will be audio-recorded and transcribed verbatim.

Data on dose and fidelity to the intervention components will also be collected using administrative data from the booking system and from the patient records.

#### Analyses

Qualitative data will be analysed using grounded theory [48]. Data analysis will be conducted with an iterative and constant comparative method, initially using an inductive approach and will be followed by deductive and abductive reasoning. The emerging analysis process will inform continued data collection. Quantitative data will be analysed using descriptive and comparative statistics. Regression methods may be used to adjust for potential baseline variables that show differences between the treatment arms at the baseline investigation.

### Ethics and dissemination

Ethical approvals have been obtained from the Swedish Ethical Review Authority, no 2022-02105-01, date of approval: 2022-06-07 and 2022-04469-02, date of approval: 2022-09-25. The study protocol approved for ethical applications can be seen in S2 and S3 Appendices. The proposed project will be conducted in accordance with the Declaration of Helsinki and oral and written consent to participate will be obtained from all participants. All study-related information about participants will be stored securely at Karolinska Institutet. Only the researchers within the group will have access to data. The results will be published in open-access, peer-reviewed journals. Dissemination will also include presentation at national and international conferences.

## Discussion

Despite the good intentions in the Swedish Health and Medical Service Act and person-centred and cohesive care process, these are not easily implemented in clinical practice. The present study will evaluate the effects of an intervention that has been developed in a co-design process including patients, significant others, and healthcare professionals, and has been further refined to fit into organizational settings and to be practical and applicable. The intervention is in agreement with the previously mentioned regulations.

The core intention of the intervention aims to create a person-centred care transition and includes a set of components that will stimulate the fulfilment of the core intentions of the intervention to improve quality of care transitions, health literacy, stroke education retention and medication adherence after stroke and TIA. The multi-component intervention targets the quality of the care transitions and support of patient health literacy through cross-organizational collaboration and pedagogical methods of information, as well as allowing for flexibility and a person-centred approach to the heterogeneous needs of this especially vulnerable group of people with stroke and TIA. The person-centred communication method Teach Back will be used in all communication intervention activities. The use of Teach Back has been shown to reduce the number of hospital readmissions and to improve medication adherence and self-management for people with chronic conditions [34, 35]. Despite positive effects, there is only one previous study of Teach Back in Sweden [50] and no studies on Teach Back in stroke care nationally or internationally. Further, health literacy is context dependent, i.e., people with high education can have low health literacy due to illness, shock, and unfamiliarity with healthcare. Projects focusing on health literacy are especially important for people with stroke and TIA due to the sudden onset, the cognitive and communicative consequences, and the risk that neglect of self-management of secondary prevention is life threatening.

## Supporting information

S1 Appendix SPIRIT 2013 checklist. (DOC)

S2 Appendix Approved study protocol by ethics committee. (PDF)

S3 Appendix Approved study protocol by ethics committee (Swedish). (PDF)
