# Supplementary material for: A person-centred care transition support for people with stroke/TIA: A study protocol for effect and process evaluation using a non-randomised controlled design
Source: PLoS One. 2024 Mar 14;19(3):e0299800. doi: 10.1371/journal.pone.0299800 (PMC10939281; doi:10.1371/journal.pone.0299800)
Supplement: S2 Appendix — (PDF) [file pone.0299800.s002.pdf]

**Approved study protocol by ethics committee:** 2022-02105-01

## Purpose and aims

The acute care of people with stroke and transient ischemic attack (TIA) is delivered in high technology stroke units with very short lengths of hospital stays. This short hospitalization means that the needed care transition from hospital care to primary care is experienced by patients as sudden and unprepared [1,2]. Swedish legislations emphasize that healthcare should be coordinated, person-centred, equal, and adapted to patients' various needs. Despite this, patients experience lack of involvement and a great responsibility to coordinate the care transition [2]. Thereto, our pre-studies have shown that the care transition is not tailored to support patients' understanding of health information needed for self-management after hospital discharge [1,2]. The national person-centred and cohesive care process for stroke and TIA emphasize the need to develop tailored communication modes to meet the various information needs of persons with stroke and TIA [3]. From an equality perspective, it is crucial that all patients regardless of communicative skills and cognitive capacity not only receive but also understand the health information. Therefore, this project has operationalized the intention of the Patient Law, the recommendations of the national person-centred and cohesive care process for stroke, and recommendations for secondary prevention. Together with users we have co-designed a multi-component care transition intervention to improve satisfaction with care transitions and understanding of health information for older people with stroke and TIA. The intervention is tailored to the needs of the most vulnerable persons, i.e., persons with communicative and cognitive barriers.

**The overall aim** is to implement and evaluate the co-designed multicomponent care transition intervention. We hypothesise that a coordinated care transition tailored to support understanding of health information will improve patient satisfaction with care transitions and patient self-management after hospital discharge, which in turn will reduce recurrent strokes/TIA and healthcare utilization.

Research questions (RQ):

1: Does a multi-component care transition intervention have an effect on functioning, perceived quality with care transitions, health literacy, medication adherence, perceived person-centeredness, recurrent stroke/TIA, healthcare utilization, and collected medications?

2. What are the patients', significant others', and healthcare professionals' experiences of the intervention components and the implementation process?

3. What contextual factors and mechanisms of the intervention can likely explain the potential effects of the intervention?

## Theoretical framework

As this co-designed multi-component care transition intervention includes *coordination of care* with focus on patient *understanding of health information*, we rely on two different frameworks: person-centred integrated care and social cognitive theory.

The project adheres to the person-centred Rainbow model of integrated care [4], on micro and meso level. Integrated care entails that the intervention is based on the individual's preferences, needs, and values, taking patient resources into consideration, i.e., is person-centred [5]. This model emphasizes the need for both functional and normative links between patients and healthcare professionals, as well as within and between hospitals and the

primary care organizations. Functional here refers to the information systems linking the hospital and primary care organizations together. Normative here refers to shared understanding and values, both within and between organizations, including taking patients' various needs and perspectives into consideration.

Health literacy emerges from theories of social exchange and social cognitive theory [6]). Social cognitive theory in turn was first known as social learning theory focusing on the principles of learning within social contexts; through the addition on "cognition" a focus on human information processing capacities that influence learning was recognized [7]. Social cognitive theory emphasizes the dynamic interplay between personal, behavioral, and environmental influences [7]. An individual's capacity to manage one's health depends on e.g., language, trust, and context of the communication situation. Effective communication is therefore crucial to enable patients to make informed decisions regarding their health condition and self-management. In this project, we will draw upon social cognitive theory in our focus on the interplay between patients and healthcare professionals for patient information processing capacity. Following social cognitive theory, we conclude that patients' self-management of secondary preventive medication is dependent on the individual professionals' communication skills.

## Background

The present project focuses on people who recently have had a stroke or TIA as: 1) they represent a large group, 2) the majority are older people, living at home, who are especially vulnerable in care transitions, 3) the disabilities that follow a stroke such as cognitive and communicative barriers can make handling information particularly challenging.

Stroke is a life-threating condition that needs acute treatment [8]. Strong evidence has shown that stroke care should initially be supplied in hospital stroke units [9]. Sweden is considered to have an efficient medical acute stroke care [10] with very short hospitalizations. Thus, the stroke chain of care always implies a care transition from inpatient care to primary care, i.e., a shift in responsibility from one healthcare setting to another.

Uncoordinated care transitions impose a burden to patients and their significant others, especially when patients and significant others lack information on how to navigate the healthcare system [8]. Cognitive impairments, post-stroke fatigue and depression [11], as well as the sudden onset of stroke and TIA, often render the patient and their significant others unprepared for the care transition to their home [12,13]. In addition, the short hospital stay leaves little time to participate in transition planning [1,2], leading to a sense of being abandoned to a new and complex life situation after discharge [2]. Swedish law requires care providers to coordinate care. Nevertheless, a great responsibility during transitions is put on the patient and significant others to coordinate the care transition [14]. Few arrangements are offered to support the patient's own ability to participate in their care or to self-manage after discharge [15,16].

There is a gap between the information provided by healthcare professionals and the information patients understand and can apply. Although 90% of recently discharged older people stated that they had understood the discharge information, 40% could not recall their diagnosis [17], 62% their new medications [18] and 23% their healthcare plan [17]. As all persons who have had a stroke receive new or revised medications, patient understanding is required not only about *what* new medications they have received, but also on *why* and *how* these medications are to be taken and *which* care provider that is responsible for medication follow-up [8,19]. Such medication management is one key task in patient self-management [20]. Self-management of secondary stroke preventive medications is crucial, as recurrent strokes account for 21% of all strokes [19] and stroke is the leading cause of disability [21]. Despite the importance of continuous medication adherence, the medical follow-up does not occur until several months after discharge [19]. This means that if a person did not understand the information at hospital, they are at risk of medication non-adherence and of getting a new

stroke. Hence, in each patient-provider meeting at hospital, it is critical to ensure that patients understand the health information to enable self-management of medications.

Patient understanding of information needed for self-management means a person's "ability to gain access to, understand and use information in ways that promote and maintain good health" [22]. This ability has been defined by WHO as health literacy [22]. WHO emphasizes that health literacy do not only depend on the individuals' ability but equally important on the capacity of healthcare organizations to provide services that support the patients' ability [23]. Low health literacy which is common after stroke [24] is associated with reduced adherence to medical advice, greater healthcare utilization, and among older persons overall poorer health status and higher mortality [25]. Thus, it has been strongly suggested that to enhance self-management after stroke healthcare services should be tailored to meet patients' varying levels of health literacy [26]. To improve patients' self-management of secondary prevention, a recent systematic review concluded that "future research should focus on the development of more effective interventions to translate secondary prevention recommendations into practice" [27].

Therefore, this project translates the intention of the Patient Law; the recommendations of the national person-centred and cohesive care process for stroke and TIA; and recommendations for secondary prevention into a multicomponent intervention co-designed to fit in practice and tailored to patient needs.

**The intervention**

The intervention has been co-designed by the users of this intervention, i.e., patients, significant others, and healthcare professionals. We also based the intervention on results from our pre-studies [1,2] and literature [28-30]. The intervention was developed with the intention to be realistic regarding what can be provided by the healthcare professionals in the involved organisational settings while focusing on patient perspective.

This multi-component intervention targets how healthcare professionals can improve quality with care transition and support health literacy for self-management of secondary prevention for persons who are to be discharged from hospitals after stroke and TIA. The intervention includes communication of "what matters to me", i.e., what is important for the individual patient; and a *bridging e-meeting* to prepare patients for home-coming. The bridging e-meeting before discharge includes the patient, their significant other, professionals at hospital and the home-rehabilitation teams in primary care (neuroteam). The intervention also includes various pedagogical modes of *information*; and a structured discharge letter on secondary preventive medications, the health condition, and plans for follow-ups at the *discharge encounter*. In all patient-provider communication (i.e., including provision of information), the person-centred communication method "Teach back" [31,32] is applied to ensure patient understanding of information; a structured communication channel between hospital staff and neuroteams.

## Preliminary and previous results

The project is a continuation of a since 2016 ongoing project, funded by Kamprad Foundation, Neuro Sweden, and the Swedish Stroke Association. In these pre-studies, we explored current state of procedures and processes during care transitions to home; needs and experiences by people with stroke, significant others, and healthcare professionals [1,2]; and patients' and significant others' outcomes (unpublished). Based on these results, we conducted a co-design process [33] in which we developed the multi-component care transition intervention presented in Figure 1 above. The intervention has been tested in a feasibility study and assessed as feasible to conduct.

In the pre-studies and in the feasibility testing, we used the same methods of inclusion of patients and significant others, and of data collection as proposed in this application. We are therefore confident that the proposed methods are well suitable for the patient and significant other population.

## Significance and scientific novelty

Despite the good intentions in the Patient Law and the person-centred and cohesive care process, these are not easily implemented in clinical practice. The here proposed intervention is an operationalization of these regulations that has been developed in a co-design process including patients, significant others, and professionals at Danderyd hospital and in primary care rehabilitation teams (neuroteams). The intervention has been further refined together with the professionals for one year to be tailored to fit into organizational settings and to be practical and applicable. The co-design process was purposefully employed to make sure that the intervention being developed accurately reflected the priorities and opinions of the targeted user group.

The originality of this proposal is that the intervention covers both functional and normative links [4] of the care transition, i.e., is multi-component, to improve satisfaction with care transitions, health literacy, and medication adherence after stroke and TIA. The multi-component intervention targets communication between patients and professionals, and between care units, as well as allowing for flexibility and person-centered approach to the heterogenous needs of this especially vulnerable group of people. It aims to increase quality with care transitions and support patient health literacy through cross-organizational collaboration and pedagogical methods of information. In all intervention components, the person-centred communication method Teach back will be used. The use of Teach Back has been shown to reduce number of hospital readmissions and improve medication adherence and self-management for people with chronic conditions [31,32]. Despite positive effects, there is only one previous study of Teach Back in Sweden [34] and no studies on Teach Back in stroke care nationally or internationally. Further, health literacy is context dependent, i.e., also persons with high levels of education can have low health literacy due to illness, shock, and unfamiliarity with healthcare. Projects focusing on health literacy is hence especially important for older people with stroke due to the sudden onset, the cognitive and communicative consequences, and as neglect of self-management of secondary prevention is life threatening.

## Methods

The project adheres to the Medical Research Council framework of complex interventions [35], which calls for phased and iterative approaches in the design and evaluation. The intervention was developed based on our pre-studies [1,2], care transition literature [28-30], and in a co-design process [36].

We will apply a non-randomized controlled trial design, which is well suited for complex interventions to assess intervention effectiveness. Randomization on patient level is not suitable, due to the risk of contamination. We have assessed that professionals in the same unit cannot separate and use different approaches for control and intervention patients. Further, the use of a non-randomized controlled trial design makes it possible to consider the role of context and the complexity of transitional care during implementation and in the evaluation. With complexity of transitional care, we acknowledge that the intervention involves communication and interaction between different professions in multiple settings (different wards at different hospitals and neuroteams of various sizes and organizational forms), as well as persons with various needs. Such complexity, defined as a "dynamic and constantly emerging set of processes and objects that not only interact with each other, but come to be defined by those interactions" [37] calls for new approaches to evaluate impact. We conclude that the complex, dynamic system of care transitions has outgrown the use of conventional scientific methods and we must apply methods that best deal with uncertainty, unpredictability, and generative causality [38]. We will therefore use a research design of one intervention site and one control site, including multiple follow-ups over one year [39] and in two phases focus on evaluation of effect and on process evaluation.

## PHASE 1: EVALUATION OF EFFECT

**RQ1:** Does a multi-component care transition intervention have an effect on functioning, perceived quality with care transitions, health literacy, medication adherence, perceived person-centeredness, recurrent stroke/TIA, healthcare utilization, and collected medications?

***Design***
Non-randomized controlled trial. The intervention will be implemented at geriatric stroke wards and acute stroke units at one hospital, and the corresponding neuroteams in primary care. Geriatric stroke wards and acute stroke units at one other hospital will serve as controls.

***Participants***
Patients: We will include patients who have had a first time ever or recurrent stroke or TIA; who are to be discharged from the participating hospitals to home and referred to a neuroteam for continued rehabilitation; and who are able to give informed consent by themselves. The patients will be informed of the study and invited to participate at the hospitals by a research assistant. The research assistant will provide oral and written information about the study and obtain consent to participate.

Significant others. Significant others will be included via the included patients. The included patients will be asked about their willingness to name a significant other for invitation to also participate in the study. The significant others will be mailed written information about the study including an informed consent and a pre-stamped envelope. Significant others who return a signed consent will be included in the study. Patients who do not have or do not want to name a significant other, will remain included in the study without a significant other.

***Data collection***
The data will be collected using questionnaires through a study specific protocol.

Patient data:
Baseline: After written consent, sociodemographic and disease-related data (e.g., stroke severity, co-morbidities) will be collected from hospital records and questionnaires. Data on patient functioning will be collected in structured interviews. Cognitive function will be assessed using Montreal Cognitive Assessment (MOCA), depression using Patient Health

Questionnaire (PHQ-2), activities of daily living using Barthel Index, recovery after stroke using a visual analogue scale, level of disability using modified Ranking Scale (mRS), and walking ability using a single-item question. This baseline data will be collected by the data collectors/research assistant.

One-two weeks after discharge, data will be collected on the primary outcome quality with care transitions using the Care Transition Measure (CTM). We will also assess health literacy using the health literacy questionnaire (HLQ), the Stroke Patient Education Retention questionnaire (SPER) and health literacy survey (HLS), medication adherence using the Medication Adherence Report Scale (MARS), perceived person-centeredness using the General person-centered care questionnaire (GPCC-Q), patient activation using the Patient activation measure (PAM), fatigue using a visual analogue scale, depression using PHQ-2, stroke severity using the modified ranking scale (mRS); activities of daily living using Barthel Index, and recovery after stroke. We will thereto ask patients about any received new or changed prescribed medications after discharge; and perceived met needs of care and rehabilitation.

At 3 and 12 months, data will be collected on health literacy using HLQ, HLS, and SPER, medication adherence using the Medication Adherence Report Scale (MARS), perceived person-centeredness using the General person-centered care questionnaire (GPCC-Q), patient activation using the Patient activation measure (PAM), fatigue using a visual analogue scale, depression using PHQ-2, stroke severity using the modified ranking scale (mRS); activities of daily living using Barthel Index, recovery after stroke, and cognitive function using MOCA. We will thereto ask patients about any received new or changed prescribed medications after discharge; and perceived met needs of care and rehabilitation.

At 12 months, data on number of recurrent strokes, unplanned hospitalizations and collected medications during the first year after stroke from Region Stockholm's database (VAL) will be collected.

Three measures of health literacy will be used to allow for psychometric evaluation.

<u>Significant other data:</u> After written consent, sociodemographic data, relation to the patient, home-help service, life satisfaction, information received, informal care supplied, and self-rated health will be collected through a study specific protocol; and data on caregiver burden[40], and health literacy using the HLQ using questionnaires in structured interviews. Data will be collected at 1-4 weeks, 3 and 12 months post patient discharge.

***Sample size***
Results from our pre-studies show that participants had a mean of 62 points in the care transition measure and standard deviation 21. Based on the estimation that satisfaction will be a mean of 72 in the new developed multi-component care transition we will need to recruit 70 patients (80% power, p=0.05, 2 sided). Allowing for 20% drop-out we will need to recruit in total 84 patients per group. Allowing for the intervention being conducted at two sites per hospital, we may need 168 patients per group, i.e., 336.

***Analyses***
Patients in the intervention group will be compared to control group using intention-to-treat and per-protocol analysis. Regression models adjusting for covariates (e.g., age, gender, stroke severity, and other disease-related data) will be used for analyses of primary and secondary outcomes.

## PHASE 2: PROCESS EVALUATION

The overall aim of the process evaluation is to explore contextual factors, implementation aspects, and mechanisms of impact that might explain the potential effects of the multi-component care transition intervention.

**RQ2:** What are the patients', significant others', and healthcare professionals' experiences of the care transition, the intervention components, and the implementation process?

**RQ3:** What contextual factors and mechanisms of the intervention can likely explain the potential effects of the intervention?

***Design***

Mixed-method process evaluation.

***Participants***

Qualitative interviews: We will use a purposive sampling for patients and significant others (e.g., age, sex, patient stroke severity, socioeconomic status) and healthcare professionals (e.g., age, sex, profession).

Observations: We will use a purposive sampling for patients and significant others (e.g., age, sex, patient stroke severity, socioeconomic status) and healthcare professionals (e.g., age, sex, profession).

Quantitative data: Healthcare professionals at the intervention sites will be asked to participate in data collection on implementation and fidelity to the intervention.

***Data collection***

Patients and significant others: Semi-structured interviews with open-ended question will be held with patients and significant others. Patient and significant other interviews will target their experience of the care transition, including how they understood information and their perspectives on interaction with healthcare professionals, and self-management at home. For patients at intervention sites, interviews will also target intervention components.

Healthcare professionals: Semi-structured interviews with open-ended question will be conducted. The healthcare professionals will be asked to describe experiences of the care transition; and for healthcare professionals in intervention sites the use of the intervention in everyday clinical practice including implementation. Data from professionals on the process of implementation and maintenance of the intervention will be collected with the Normalization MeAsure Development (NoMAD), which is based on the implementation theory Normalization Process Theory [41]. NoMAD will be iteratively assessed from intervention start to monitor implementation of the intervention. Data on dose and fidelity to the intervention components will be collected on use of intervention components in clinical practice using professionals' self-reports.

Patients, significant others, and healthcare professionals: The interaction between patients, significant others, and healthcare professionals will be observed at hospital and during neuroteams encounters at home.

All interviews and observations will be audio-recorded and transcribed verbatim.

Data on dose and fidelity to the intervention components will also be collected using administrative data from booking system and patient records.

### *Analyses*

Qualitative data will be analysed using qualitative content analysis. Quantitative data will be analysed using descriptive and comparative statistics.

## References


1. Lindblom S, Flink M, Sjöstrand S, Laska A, von Koch L, Ytterberg C. Perceived Quality of Care Transitions between Hospital and the Home in People with Stroke. *J Am Med Dir Assoc.* 2020.
2. Lindblom S, Ytterberg C, Elf M, Flink M. Perceptive Dialogue for Linking Stakeholders and Units During Care Transitions - A Qualitative Study of People with Stroke, Significant Others and Healthcare Professionals in Sweden. *Int J Integr Care.* 2020;20(1):11.
3. Personcentrerade sammanhållna vårdförlopp [Person-centred and cohesive care process]. https://kunskapsstyrningvard.se/kunskapsstod/personcentreradesammanhallnavardforlopp.834.html. Accessed 10 June, 2021.
4. Valentijn PP. Rainbow of Chaos: A study into the Theory and Practice of Integrated Primary Care: Pim P. Valentijn, [S.l.: s.n.], 2015 (Print Service Ede), pp. 195, Doctoral Thesis Tilburg University, The Netherlands, ISBN: 978-94-91602-40-5. *Int J Integr Care.* 2016;16(2):3.
5. Institute_of_Medicine. *Crossing the quality chasm: a new health system for the twenty-first century.* Washington: National Academies Press;2001.
6. Hepburn M. Health Literacy, Conceptual Analysis for Disease Prevention. *International Journal of Collaborative Research on Internal Medicine & Public Health & Social Care in the Community.* 2012;4(3):11.
7. McAlister A, Perry C, Parcel G. How individuals, environments, and health behaviors interact - Social Cognitive Theory. In: Glanz K, Rimer N, Viswanath K, eds. *Health behavior and health education.* San Fransisco, USA: Jossey-Bass.
8. Wissel J, Olver J, Sunnerhagen KS. Navigating the poststroke continuum of care. *Journal of stroke and cerebrovascular diseases : the official journal of National Stroke Association.* 2013;22(1):1-8.
9. Langhorne P, Ramachandra S. Organised inpatient (stroke unit) care for stroke: network meta-analysis. *Cochrane Database Syst Rev.* 2020;4:Cd000197.
10. OECD. *Sweden: Country Health Profile 2019.* OECD Publishing, Paris/European Observatory on Health Systems and Policies, Brussels2019.
11. Aarnes R, Stubberud J, Lerdal A. A literature review of factors associated with fatigue after stroke and a proposal for a framework for clinical utility. *Neuropsychological rehabilitation.* 2019:1-28.
12. Connolly T, Mahoney E. Stroke survivors' experiences transitioning from hospital to home. *J Clin Nurs.* 2018;27(21-22):3979-3987.
13. Wottrich AW, Astrom K, Lofgren M. On parallel tracks: newly home from hospital--people with stroke describe their expectations. *Disability and rehabilitation.* 2012;34(14):1218-1224.
14. The_Swedish_Agency_for_Health_and_Care_Services_Analysis. *Coordinated health and care services An analysis of the coordination challenges in a fragmented system for health and care services.* Stockholm2016.
15. Flink M, Ekstedt M. Planning for the Discharge, not for Patient Self-Management at Home - An Observational and Interview Study of Hospital Discharge. *Int J Integr Care.* 2017;17(6):1.
16. Gustafsson L, Bootle K. Client and carer experience of transition home from inpatient stroke rehabilitation. *Disability and rehabilitation.* 2013;35(16):1380-1386.
17. Lin MJ, Tirosh AG, Landry A. Examining patient comprehension of emergency department discharge instructions: Who says they understand when they do not? *Intern Emerg Med.* 2015;10(8):993-1002.

18. Ziaeian B, Araujo KL, Van Ness PH, Horwitz LI. Medication reconciliation accuracy and patient understanding of intended medication changes on hospital discharge. *Journal of general internal medicine.* 2012;27(11):1513-1520.
19. Riks-Stroke. *Stroke och TIA - ÅRSRAPPORT FRÅN RIKSSTROKE.* 2019.
20. Lorig KR, Holman H. Self-management education: history, definition, outcomes, and mechanisms. *Annals of behavioral medicine : a publication of the Society of Behavioral Medicine.* 2003;26(1):1-7.
21. Global, regional, and national burden of stroke, 1990-2016: a systematic analysis for the Global Burden of Disease Study 2016. *Lancet Neurol.* 2019;18(5):439-458.
22. World_Health_Organization. *Health Promotion Glossary.* Geneva1998.
23. Osborne RH, Batterham RW, Elsworth GR, Hawkins M, Buchbinder R. The grounded psychometric development and initial validation of the Health Literacy Questionnaire (HLQ). *BMC Public Health.* 2013;13:658.
24. Hoffmann T, McKenna K. Analysis of stroke patients' and carers' reading ability and the content and design of written materials: recommendations for improving written stroke information. *Patient Educ Couns.* 2006;60(3):286-293.
25. Berkman ND, Sheridan SL, Donahue KE, Halpern DJ, Crotty K. Low health literacy and health outcomes: an updated systematic review. *Annals of internal medicine.* 2011;155(2):97-107.
26. Aaby A, Friis K, Christensen B, Rowlands G, Maindal HT. Health literacy is associated with health behaviour and self-reported health: A large population-based study in individuals with cardiovascular disease. *Eur J Prev Cardiol.* 2017;24(17):1880-1888.
27. Bridgwood B, Lager KE, Mistri AK, Khunti K, Wilson AD, Modi P. Interventions for improving modifiable risk factor control in the secondary prevention of stroke. *Cochrane Database Syst Rev.* 2018;5:CD009103.
28. Goncalves-Bradley DC, Lannin NA, Clemson LM, Cameron ID, Shepperd S. Discharge planning from hospital. *Cochrane Database Syst Rev.* 2016(1):Cd000313.
29. Langhorne P, Baylan S. Early supported discharge services for people with acute stroke. *Cochrane Database Syst Rev.* 2017;7:Cd000443.
30. Leppin AL, Gionfriddo MR, Kessler M, et al. Preventing 30-Day Hospital Readmissions: A Systematic Review and Meta-analysis of Randomized Trials. *JAMA internal medicine.* 2014.
31. Ha Dinh TT, Bonner A, Clark R, Ramsbotham J, Hines S. The effectiveness of the teach-back method on adherence and self-management in health education for people with chronic disease: a systematic review. *JBI Database System Rev Implement Rep.* 2016;14(1):210-247.
32. Oh EG, Lee HJ, Yang YL, Kim YM. Effectiveness of Discharge Education With the Teach-Back Method on 30-Day Readmission: A Systematic Review. *J Patient Saf.* 2019.
33. Lindblom S, Flink M, Elf M, Laska AC, von Koch L, Ytterberg C. The manifestation of participation within a co-design process involving patients, significant others and health-care professionals. *Health Expect.* 2021.
34. Berthelsen O, Wiklund M, Säthil K, Samulowitz A, Fagevik Olsén M. An evaluation of two different methods for preoperative physical therapy information before abdominal surgery. *Journal of Communication in Healthcare.* 2020;13(2):102-110.
35. Craig P, Dieppe P, Macintyre S, Michie S, Nazareth I, Petticrew M. Developing and evaluating complex interventions: the new Medical Research Council guidance. *Bmj.* 2008;337:a1655.
36. Boyd H, McKernon S, Mullin B, Old A. Improving healthcare through the use of co-design. *N Z Med J.* 2012;125(1357):76-87.
37. Cohn S, Clinch M, Bunn C, Stronge P. Entangled complexity: why complex interventions are just not complicated enough. *J Health Serv Res Policy.* 2013;18(1):40-43.
38. Greenhalgh T, Papoutsi C. Studying complexity in health services research: desperately seeking an overdue paradigm shift. *BMC Med.* 2018;16(1):95.
39. Moore GF, Evans RE, Hawkins J, et al. From complex social interventions to interventions in complex social systems: Future directions and unresolved questions for intervention development and evaluation. *Evaluation (Lond).* 2019;25(1):23-45.

40. Elmstahl S, Malmberg B, Annerstedt L. Caregiver's burden of patients 3 years after stroke assessed by a novel caregiver burden scale. *Archives of physical medicine and rehabilitation.* 1996;77(2):177-182.
41. Elf M, Nordmark S, Lyhagen J, Lindberg I, Finch T, Aberg AC. The Swedish version of the Normalization Process Theory Measure S-NoMAD: translation, adaptation, and pilot testing. *Implementation science : IS.* 2018;13(1):146.
