# Supplementary material for: A person-centred care transition support for people with stroke/TIA: A study protocol for effect and process evaluation using a non-randomised controlled design
Source: PLoS One. 2024 Mar 14;19(3):e0299800. doi: 10.1371/journal.pone.0299800 (PMC10939281; doi:10.1371/journal.pone.0299800)
Supplement: S3 Appendix — (PDF) [file pone.0299800.s003.pdf]

Godkänd studieprotokoll, etikprövningsnämnden: 2022-02105-01

Syfte och frågeställningar
Den akuta vården av personer med stroke och transient ischemisk attack (TIA) ges på högteknologiska strokeenheter med mycket korta sjukhusvistelser. Denna korta vårdtiden innebär att den nödvändiga vårdövergången från sjukhusvård till primärvård upplevs av patienter som plötslig och oförberedd (1,2). Svensk lagstiftning betonar att vården ska vara samordnad, personcentrerad, jämlik och anpassad till patienternas olika behov. Trots detta upplever patienterna bristande engagemang och ett stort ansvar för att samordna vårdövergången (2). Därtill har våra förstudier visat att vårdövergången inte är skräddarsydd för att stödja patienters förståelse av hälsoinformation som behövs för egenvård efter sjukhusutskrivning (1,2). Den nationella personcentrerade och sammanhållna vårdprocessen för stroke och TIA betonar behovet av att utveckla skräddarsydda kommunikationssätt för att möta de olika informationsbehoven hos personer med stroke och TIA (3). Ur ett jämlikhetsperspektiv är det avgörande att alla patienter oavsett kommunikativ kompetens och kognitiv kapacitet inte bara ta emot utan också förstå hälsoinformationen. Därför har detta projekt operationaliserat patientlagens avsikt, rekommendationerna från den nationella personcentrerade och sammanhållna vårdprocessen för stroke och rekommendationer för sekundärprevention. Tillsammans med användare har vi designat en multikomponent vårdövergångsintervention för att förbättra tillfredsställelsen med vårdövergångar och förståelse för hälsoinformation för äldre personer med stroke och TIA. Insatsen är skräddarsydd efter behoven hos de mest utsatta personerna, det vill säga personer med kommunikativa och kognitiva barriärer.

Det övergripande målet är att implementera och utvärdera den samdesignade multikomponentvårdsövergångsinterventionen. Vi antar att en samordnad vårdövergång skräddarsydd för att stödja förståelsen av hälsoinformation kommer att förbättra patienternas tillfredsställelse med vårdövergångar och patientens självhantering efter sjukhusutskrivning, vilket i sin tur kommer att minska återkommande stroke/TIA och sjukvårdsanvändning.

Det övergripande syftet är att implementera och utvärdera en samskapad vårdövergång bestående av flera komponenter

Frågeställningar:

1: Vilken effekt har den nya vårdövergången på patienters funktionstillstånd, upplevelse av vårdövergångens kvalitet, förståelse av hälsoinformation, följsamhet till läkemedelsbehandling, upplevelse av person-centrering, uthämtade läkemedel, återinsjuknande i stroke/TIA, hälso- och sjukvårdsanvändning?

2. Vilka erfarenheter har patienter, närstående och hälso- och sjukvårdspersonal av vårdövergången?

3. Vilka möjliga mekanismer och kontextuella faktorer kan förklara eventuella effekter av interventionen?

Teoretiskt ramverk

Eftersom denna samdesignade multi-komponent vårdövergångsintervention inkluderar koordinering av vården med fokus på patientens förståelse av hälsoinformation, förlitar vi oss på två olika ramar: personcentrerad integrerad vård och social kognitiv teori.

Projektet följer den personcentrerade Rainbow-modellen för integrerad vård (4), på mikro- och mesonivå. Integrerad vård innebär att insatsen utgår från individens preferenser, behov

och värderingar, med hänsyn till patientresurser, dvs är personcentrerad (5). Denna modell betonar behovet av både funktionella och normativa kopplingar mellan patienter och vårdpersonal, eftersom såväl som inom och mellan sjukhus och primärvårdens organisationer. Funktionell avser här de informationssystem som länkar samman sjukhus och primärvårdsorganisationer. Normativ avser här delad förståelse och värderingar, både inom och mellan organisationer, inklusive att ta hänsyn till patienters olika behov och perspektiv.

Hälsolitteracitet kommer från teorier om socialt utbyte och social kognitiv teori (6). Social kognitiv teori i sin tur var först känd som social inlärningsteori med fokus på principerna för lärande inom sociala sammanhang; genom tillägget om "kognition" erkändes ett fokus på mänsklig informationsbehandlingskapacitet som påverkar lärande 7. Socialkognitiv teori betonar det dynamiska samspelet mellan personliga, beteendemässiga och miljömässiga influenser (7). En individs förmåga att hantera sin hälsa beror på t.ex. språk, förtroende och sammanhang i kommunikationssituationen. Effektiv kommunikation är därför avgörande för att patienter ska kunna fatta välgrundade beslut angående sitt hälsotillstånd och självhantering. I detta projekt kommer vi att dra nytta av social kognitiv teori i vårt fokus på samspelet mellan patienter och vårdpersonal för patientinformationsbearbetningskapacitet. Efter socialkognitiv teori drar vi slutsatsen att patienternas självhantering av sekundärpreventiv medicinering är beroende av de individuella yrkesutövarnas kommunikationsförmåga.

Background

Det aktuella projektet fokuserar på personer som nyligen har haft en stroke eller TIA eftersom: 1) de representerar en stor grupp, 2) majoriteten är äldre personer, som bor hemma, som är särskilt utsatta i vårdövergångar, 3) de funktionsnedsättningar som följer en stroke som kognitiva och kommunikativa hinder kan göra hanteringen av information särskilt utmanande.

Stroke är ett livshotande tillstånd som kräver akut behandling (8). Stark evidens har visat att strokevård initialt bör ges på strokeenheter på sjukhus (9). Sverige anses ha en effektiv medicinsk akut strokevård (10) med mycket korta sjukhusvistelser. Vårdkedjan för stroke innebär alltså alltid en vårdövergång från slutenvård till primärvård, det vill säga en förskjutning av ansvar från en vårdmiljö till en annan.

Okoordinerade vårdövergångar medför en börda för patienter och deras betydelsefulla andra, särskilt när patienter och betydelsefulla andra saknar information om hur de ska navigera i sjukvården (8). Kognitiva funktionsnedsättningar, trötthet efter stroke och depression (11), samt plötsligt debut av stroke och TIA, gör ofta patienten och deras betydelsefulla andra oförberedda för vårdövergången till sitt hem (12,13). Dessutom ger den korta sjukhusvistelsen lite tid att delta i övergångsplanering (1,2), vilket leder till en känsla av att vara övergiven till en ny och komplex livssituation efter utskrivning (2). Svensk lag kräver att vårdgivare samordnar vården. Ändå läggs ett stort ansvar vid övergångar på patienten och betydande andra att samordna vårdövergången (14). Få arrangemang erbjuds för att stödja patientens egen förmåga att delta i sin vård eller att klara sig själv efter utskrivning (15,16).

Det finns en lucka mellan den information som tillhandahålls av vårdpersonal och den information som patienterna förstår och kan tillämpa. Även om 90 % av nyligen utskrivna äldre uppgav att de hade förstått utskrivningsinformationen, kunde 40 % inte komma ihåg sin diagnos (17), 62 % sina nya mediciner (18) och 23 % sin vårdplan (17). Eftersom alla personer som har haft en stroke får nya eller reviderade mediciner krävs patientförståelse inte bara om vilka nya mediciner de har fått utan även om varför och hur dessa mediciner ska tas och vilken vårdgivare som ansvarar för medicinuppföljning (8,19). Sådan läkemedelshantering är en nyckeluppgift i patientens självhantering (20). Självhantering av sekundära strokeförebyggande mediciner är avgörande, eftersom återkommande stroke står

för 21 % av alla stroke (19) och stroke är den främsta orsaken till funktionshinder (21). Trots vikten av kontinuerlig medicinföljning sker den medicinska uppföljningen inte förrän flera månader efter utskrivning (19). Det innebär att om en person inte förstått informationen på sjukhuset riskerar man att brista i följsamhet till läkemedel och att få en ny stroke. Därför är det viktigt att försäkra sig om att patienterna förstår hälsoinformationen vid varje möte mellan patient och sjukvårdspersonal på sjukhuset för att möjliggöra egenvård av mediciner.

Patienternas förståelse för information som behövs för självförvaltning innebär en persons "förmåga att få tillgång till, förstå och använda information på ett sätt som främjar och upprätthåller god hälsa" (22). Denna förmåga har definierats av WHO som hälsoläskunnighet 22. WHO betonar att hälsa läskunnighet beror inte bara på individernas förmåga utan lika viktigt på sjukvårdsorganisationernas förmåga att tillhandahålla tjänster som stödjer patienternas förmåga (23). Låg läskunnighet som är vanlig efter stroke (24) är förknippad med minskad följsamhet till medicinsk rådgivning, ökad sjukvård utnyttjande och bland äldre personer totalt sett sämre hälsotillstånd och högre dödlighet (25). Det har därför starkt föreslagits att sjukvården för att förbättra självförvaltningen efter stroke bör skräddarsys för att möta patienters varierande nivåer av hälsokompetens (26). För att förbättra patienternas självförvaltning av sekundärt förebyggande, drog en nyligen genomförd systematisk granskning slutsatsen att "framtida forskning bör fokusera på utvecklingen av mer effektiva insatser för att omsätta rekommendationer för sekundärprevention i praktiken" (27).

Därför översätter detta projekt patientlagens avsikt; rekommendationerna från den nationella personcentrerade och sammanhållna vårdprocessen för stroke och TIA; och rekommendationer för sekundär prevention till en multikomponentintervention som är samdesignad för att passa i praktiken och skräddarsys för patientens behov.

Interventionen

Interventionen har utformats tillsammans av användarna av denna intervention, det vill säga patienter, betydande andra och vårdpersonal. Vi baserade även interventionen på resultat från våra förstudier (1,2) och litteratur (28-30). Interventionen utvecklades med avsikten att vara realistisk när det gäller vad som kan tillhandahållas av vårdpersonal i de involverade organisatoriska miljöerna samtidigt som patientperspektivet fokuseras.

Denna multikomponentintervention är inriktad på hur vårdpersonal kan förbättra kvaliteten med vårdövergång och stödja hälsokompetens för självhantering av sekundärprevention för personer som ska skrivas ut från sjukhus efter stroke och TIA. Interventionen innefattar kommunikation av ”vad som betyder något för mig”, det vill säga vad som är viktigt för den enskilda patienten; och ett överbryggande e-möte för att förbereda patienter för hemkomst. Det överbryggande e-mötet före utskrivning inkluderar patienten, dennes närastående, yrkesverksamma på sjukhuset och hemrehabiliteringsteamen i primärvården (neuroteam). Interventionen omfattar även olika pedagogiska informationssätt; och ett strukturerat utskrivningsbrev om sekundära förebyggande mediciner, hälsotillståndet och planer för uppföljning vid utskrivningsmötet. I all kommunikation mellan patient och leverantör (dvs. inklusive tillhandahållande av information) tillämpas den personcentrerade kommunikationsmetoden "Teach back" (31,32) för att säkerställa att patienten förstår informationen; en strukturerad kommunikationskanal mellan sjukhuspersonal och neuroteam.

Preliminary and previous results

Projektet är en fortsättning på ett sedan 2016 pågående projekt, finansierat av Kampradstiftelsen, Neuro Sweden och Svenska Strokeförbundet. I dessa förstudier undersökte vi nuvarande tillstånd för procedurer och processer under vårdövergångar till hemmet; behov och upplevelser av personer med stroke, betydande andra och vårdpersonal (1,2); och patienters och signifikanta andras resultat (opublicerade). Baserat på dessa

resultat genomförde vi en samdesignprocess (33) där vi utvecklade den multikomponentvårdsövergångsintervention som presenteras i figur 1 ovan. Insatsen har testats i en förstudie och bedömts vara genomförbar.

I förstudierna och i genomförbarhetstesterna använde vi samma metoder för inkludering av patienter och signifikanta andra, och för datainsamling som föreslagits i denna ansökan. Vi är därför övertygade om att de föreslagna metoderna är väl lämpade för patienten och betydande annan population.

Signifikans och nyskapande

Trots de goda intentionerna i Patientlagen och den personcentrerade och sammanhållna vårdprocessen är dessa inte lätta att implementera i klinisk praxis. Den här föreslagna interventionen är en operationalisering av dessa regelverk som har utvecklats i en co-designprocess inklusive patienter, betydande andra och yrkesverksamma vid Danderyds sjukhus och i primärvårdens rehabiliteringsteam (neuroteam). Interventionen har vidareutvecklats tillsammans med yrkesverksamma under ett år för att skräddarsys för att passa in i organisatoriska miljöer och för att vara praktisk och användbar. Co-designprocessen användes målmedvetet för att säkerställa att interventionen som utvecklades korrekt återspeglade prioriteringarna och åsikterna hos den målgruppsanvändargruppen.

Originaliteten i detta förslag är att interventionen täcker både funktionella och normativa länkar (4) av vårdövergången, det vill säga är multikomponent, för att förbättra tillfredsställelsen med vårdövergångar, hälsokompetens och medicinering efter stroke och TIA. Interventionen med flera komponenter är inriktad på kommunikation mellan patienter och professionella, och mellan vårdenheter, samt möjliggör flexibilitet och personcentrerad inställning till de heterogena behoven hos denna särskilt utsatta grupp människor. Det syftar till att öka kvaliteten med vårdövergångar och stödja patienternas hälsokompetens genom tvärorganisatoriskt samarbete och pedagogiska metoder för information. I alla insatskomponenter kommer den personcentrerade kommunikationsmetoden Teach back användas. Användningen av Teach Back har visat sig minska antalet återinläggningar på sjukhus och förbättra medicinering och självhantering för personer med kroniska tillstånd (31,32). Trots positiva effekter finns det endast en tidigare studie av Teach Back i Sverige (34) och inga studier om Teach Back inom strokevården nationellt eller internationellt. Vidare är hälsokunskaper kontextberoende, det vill säga också personer med hög utbildning kan ha låg hälsokompetens på grund av sjukdom, chock och obekantskap med sjukvården. Projekt som fokuserar på hälsokompetens är därför särskilt viktiga för äldre personer med stroke på grund av plötsligt debut, kognitiva och kommunikativa konsekvenser, och eftersom försummelse av självhantering av sekundärprevention är livshotande.

Metod

Projektet följer Medical Research Councils ramverk för komplexa interventioner (35), vilket kräver stegvisa och iterativa tillvägagångssätt vid design och utvärdering. Interventionen utvecklades utifrån våra förstudier (1,2), vårdövergångslitteratur (28-30) och i en samdesignprocess (36).

Vi kommer att tillämpa en icke-randomiserad kontrollerad studiedesign, som är väl lämpad för komplexa interventioner för att bedöma interventionens effektivitet. Randomisering på patientnivå är inte lämplig på grund av risken för kontaminering. Vi har bedömt att professionella inom samma enhet inte kan separera och använda olika angreppssätt för kontroll- och interventionspatienter. Vidare gör användningen av en icke-randomiserad kontrollerad prövningsdesign det möjligt att överväga sammanhangets roll och komplexiteten i övergångsvård under implementeringen och i utvärderingen. Med komplexiteten i övergångsvården erkänner vi att interventionen involverar kommunikation och interaktion

mellan olika professioner i flera miljöer (olika avdelningar på olika sjukhus och neuroteam av olika storlekar och organisationsformer), såväl som personer med olika behov. Sådan komplexitet, definierad som en "dynamisk och ständigt framväxande uppsättning processer och objekt som inte bara interagerar med varandra, utan kommer att definieras av dessa interaktioner" (37) kräver nya tillvägagångssätt för att utvärdera påverkan. Vi drar slutsatsen att det komplexa, dynamiska systemet med vårdövergångar har vuxit ur användningen av konventionella vetenskapliga metoder och vi måste tillämpa metoder som bäst hanterar osäkerhet, oförutsägbarhet och generativ kausalitet (38). Vi kommer därför att använda en forskningsdesign av en interventionsplats och en kontrollplats, inklusive flera uppföljningar under ett år (39) och i två faser fokus på utvärdering av effekt och på processutvärdering.

FAS 1: EFFEKTUTVÄRDERING

Frågeställning 1: 1: Vilken effekt har den nya vårdövergången på patienters funktionstillstånd, upplevelse av vårdövergångens kvalitet, förståelse av hälsoinformation, följsamhet till läkemedelsbehandling, upplevelse av person-centrering, uthämtade läkemedel, återinsjuknande i stroke/TIA, hälso- och sjukvårdsanvändning?

Design
Icke-randomiserad kontrollerad studie. Insatsen kommer att genomföras på geriatriska strokeavdelningar och akuta strokeenheter på ett sjukhus, och motsvarande neuroteam inom primärvården. Geriatriska strokeavdelningar och akuta strokeenheter på ett annat sjukhus kommer att fungera som kontroller.

Deltagare
Patienter: Vi kommer att inkludera patienter som har haft en första gång någonsin eller återkommande stroke eller TIA; som ska skrivas ut från de deltagande sjukhusen till hemmet och remitteras till ett neuroteam för fortsatt rehabilitering; och som själva kan ge informerat samtycke. Patienterna kommer att informeras om studien och bjudas in att delta på sjukhusen av en forskningsassistent. Forskningsassistenten kommer att ge muntlig och skriftlig information om studien och inhämta samtycke till att delta.

Närstående: Närstående kommer att inkluderas via de inkluderade patienterna. De inkluderade patienterna kommer att tillfrågas om deras vilja att namnge en signifikant annan för inbjudan att också delta i studien. De betydelsefulla andra kommer att skickas skriftlig information om studien, inklusive ett informerat samtycke och ett i förväg stämplat kuvert. Betydande andra som returnerar ett undertecknat samtycke kommer att inkluderas i studien. Patienter som inte har eller inte vill nämna en signifikant annan, kommer att förbli inkluderade i studien utan en signifikant annan.

Datainsamling
Data kommer att samlas in genom ett studiespecifikt frågeformulär.

Patientdata:
Baslinje: Efter skriftligt medgivande kommer sociodemografiska och sjukdomsrelaterade data (t.ex. strokesvårighet, samsjuklighet) att samlas in från sjukhusjournaler och frågeformulär. Data om patientens funktion kommer att samlas in i strukturerade intervjuer. Kognitiv funktion kommer att bedömas med hjälp av Montreal Cognitive Assessment (MOCA), depression med hjälp av Patient Health Questionnaire (PHQ-2), dagliga aktiviteter med Barthel Index, återhämtning efter stroke med hjälp av en visuell analog skala, funktionsnedsättning med modifierad rankningsskala (mRS), och gångförmåga med hjälp av en fråga med en enda punkt. Dessa basdata kommer att samlas in av datainsamlarna/forskarassistenten.

En-två veckor efter utskrivningen kommer data att samlas in om den primära utfallskvaliteten med vårdövergångar med hjälp av Care Transition Measure (CTM). Vi kommer också att utvärdera hälsokunskaper med hjälp av frågeformuläret för hälsoläskunnighet (HLQ), Stroke Patient Education Retention questionnaire (SPER) och hälsoläskunnighet (HLS), medicinadherence med hjälp av Medication Adherence Report Scale (MARS), upplevd personcentrering med hjälp av Allmänt personcentrerat vårdformulär (GPCC-Q), patientaktivering med patientaktiveringsmåttet (PAM), trötthet med hjälp av en visuell analog skala, depression med PHQ-2, slaganfall med hjälp av den modifierade rankningsskalan (mRS); dagliga aktiviteter med Barthel Index och återhämtning efter stroke. Vi kommer därför att fråga patienter om eventuella mottagna nya eller ändrade ordinerade mediciner efter utskrivning; och upplevde tillgodosedda behov av vård och rehabilitering.

Efter 3 och 12 månader kommer data att samlas in om hälsokompetens med hjälp av HLQ, HLS och SPER, medicinadherence med hjälp av Medication Adherence Report Scale (MARS), upplevd personcentrering med hjälp av General person-centred care questionnaire (GPCC-Q) , patientaktivering med patientaktiveringsmåttet (PAM), trötthet med hjälp av en visuell analog skala, depression med PHQ-2, slaganfallsgrad med den modifierade rankningsskalan (mRS); dagliga aktiviteter med Barthel Index, återhämtning efter stroke och kognitiv funktion med MOCA. Vi kommer därför att fråga patienter om eventuella mottagna nya eller ändrade ordinerade mediciner efter utskrivning; och upplevde tillgodosedda behov av vård och rehabilitering.

Vid 12 månader kommer data om antal återkommande stroke, oplanerade sjukhusinläggningar och insamlade mediciner under det första året efter stroke att samlas in från Region Stockholms databas (VAL).

Tre mått på hälsokompetens kommer att användas för att möjliggöra psykometrisk utvärdering.

Närståendedata:
Efter skriftligt medgivande kommer sociodemografiska data, relation till patienten, hemtjänst, tillfredsställelse med livet, mottagen information, tillhandahållen informell vård och självskattad hälsa att samlas in genom ett studiespecifikt protokoll; och data om vårdgivares börda40 och hälsokompetens med hjälp av HLQ med hjälp av frågeformulär i strukturerade intervjuer. Data kommer att samlas in 1-4 veckor, 3 och 12 månader efter patientens utskrivning.

Powerberäkning
Resultat från våra förstudier visar att deltagarna hade ett medelvärde på 62 poäng i vårdövergångsmåttet och standardavvikelse 21. Baserat på uppskattningen att tillfredsställelsen blir ett medelvärde på 72 i den nyutvecklade flerkomponentvårdsövergången kommer vi att behöva rekrytera 70 patienter (80 % effekt, p=0,05, 2-sidig). Med 20 % avhopp kommer vi att behöva rekrytera totalt 84 patienter per grupp. Med tanke på att interventionen kan utföras på två platser per sjukhus kan vi behöva 168 patienter per grupp, dvs 336.

Analyser
Patienter i interventionsgruppen kommer att jämföras med kontrollgruppen med hjälp av intention-to-treat och per-protokollanalys. Regressionsmodeller som justerar för kovariater (t.ex. ålder, kön, svårighetsgrad av stroke och andra sjukdomsrelaterade data) kommer att användas för analyser av primära och sekundära utfall.

FAS 2: PROCESSUTVÄRDERING

Det övergripande syftet med processutvärderingen är att utforska kontextuella faktorer, implementeringsaspekter och påverkansmekanismer som kan förklara de potentiella effekterna av multikomponentvårdsövergångsinterventionen.

Frågeställning 2. Vilka erfarenheter har patienter, närstående och hälso- och sjukvårdspersonal av vårdövergången?

Frågeställning 3. Vilka möjliga mekanismer och kontextuella faktorer kan förklara eventuella effekter av interventionen?

Design
Processutvärdering med mixed-method.

Deltagare
Kvalitativa intervjuer: Vi kommer att använda ett målinriktat urval för patienter och signifikanta andra (t.ex. ålder, kön, patientens svårighetsgrad för stroke, socioekonomisk status) och vårdpersonal (t.ex. ålder, kön, yrke).

Observationer: Vi kommer att använda ett målinriktat urval för patienter och betydande andra (t.ex. ålder, kön, patientens slaganfall, socioekonomisk status) och vårdpersonal (t.ex. ålder, kön, yrke).

Kvantitativa data: Hälso- och sjukvårdspersonal på interventionsplatserna kommer att uppmanas att delta i datainsamlingen om genomförande och trohet mot interventionen.

Datainsamling

Patienter och närstående: Semistrukturerade intervjuer med öppna frågor kommer att hållas med patienter och närstående. Intervjuer med patienter och betydande andra kommer att inrikta sig på deras upplevelse av vårdövergången, inklusive hur de förstod information och deras perspektiv på interaktion med vårdpersonal och självhantering i hemmet. För patienter på interventionsplatser kommer intervjuer också att inriktas på interventionskomponenter.

Vårdpersonal:
Semistrukturerade intervjuer med öppna frågor kommer att genomföras. Vårdpersonalen kommer att få beskriva erfarenheter av vårdövergången; och för vårdpersonal på interventionsställen användningen av interventionen i den dagliga kliniska praktiken inklusive implementering. Data från professionella om processen för implementering och underhåll av interventionen kommer att samlas in med Normalization MeAsure Development (NoMAD), som är baserad på implementeringsteorin Normalization Process Theory (41). NoMAD kommer att utvärderas iterativt från start av insatsen för att övervaka implementeringen av intervention. Data om dos och trohet mot interventionskomponenterna kommer att samlas in om användningen av interventionskomponenter i klinisk praxis med hjälp av professionella självrapporter.

Patienter, närstående och vårdpersonal: Interaktionen mellan patienter, betydande andra och vårdpersonal kommer att observeras på sjukhus och under möten mellan neuroteam i hemmet.

Alla intervjuer och observationer kommer att spelas in på ljud och transkriberas ordagrant.

Data om dos och trohet mot interventionskomponenterna kommer också att samlas in med hjälp av administrativa data från bokningssystem och patientjournaler.

Analyser
Kvalitativ data kommer att analyseras med hjälp av kvalitativ innehållsanalys. Kvantitativ data kommer att analyseras med hjälp av beskrivande och jämförande statistik.

Referenser

1. Lindblom S, Flink M, Sjöstrand S, Laska A, von Koch L, Ytterberg C. Perceived Quality of Care Transitions between Hospital and the Home in People with Stroke. *J Am Med Dir Assoc.* 2020.
2. Lindblom S, Ytterberg C, Elf M, Flink M. Perceptive Dialogue for Linking Stakeholders and Units During Care Transitions - A Qualitative Study of People with Stroke, Significant Others and Healthcare Professionals in Sweden. *Int J Integr Care.* 2020;20(1):11.
3. Personcentrerade sammanhållna vårdförlopp [Person-centred and cohesive care process]. https://kunskapsstyrningvard.se/kunskapsstod/personcentreradesammanhallnavardforlopp.834.html. Accessed 10 June, 2021.
4. Valentijn PP. Rainbow of Chaos: A study into the Theory and Practice of Integrated Primary Care: Pim P. Valentijn, [S.l.: s.n.], 2015 (Print Service Ede), pp. 195, Doctoral Thesis Tilburg University, The Netherlands, ISBN: 978-94-91602-40-5. *Int J Integr Care.* 2016;16(2):3.
5. Institute_of_Medicine. *Crossing the quality chasm: a new health system for the twenty-first century.* Washington: National Academies Press;2001.
6. Hepburn M. Health Literacy, Conceptual Analysis for Disease Prevention. *International Journal of Collaborative Research on Internal Medicine & Public Health & Social Care in the Community.* 2012;4(3):11.
7. McAlister A, Perry C, Parcel G. How individuals, environments, and health behaviors interact - Social Cognitive Theory. In: Glanz K, Rimer N, Viswanath K, eds. *Health behavior and health education.* San Fransisco, USA: Jossey-Bass.
8. Wissel J, Olver J, Sunnerhagen KS. Navigating the poststroke continuum of care. *Journal of stroke and cerebrovascular diseases : the official journal of National Stroke Association.* 2013;22(1):1-8.
9. Langhorne P, Ramachandra S. Organised inpatient (stroke unit) care for stroke: network meta-analysis. *Cochrane Database Syst Rev.* 2020;4:Cd000197.
10. OECD. *Sweden: Country Health Profile 2019.* OECD Publishing, Paris/European Observatory on Health Systems and Policies, Brussels2019.
11. Aarnes R, Stubberud J, Lerdal A. A literature review of factors associated with fatigue after stroke and a proposal for a framework for clinical utility. *Neuropsychological rehabilitation.* 2019:1-28.
12. Connolly T, Mahoney E. Stroke survivors' experiences transitioning from hospital to home. *J Clin Nurs.* 2018;27(21-22):3979-3987.
13. Wottrich AW, Astrom K, Lofgren M. On parallel tracks: newly home from hospital--people with stroke describe their expectations. *Disability and rehabilitation.* 2012;34(14):1218-1224.
14. The_Swedish_Agency_for_Health_and_Care_Services_Analysis. *Coordinated health and care services An analysis of the coordination challenges in a fragmented system for health and care services.* Stockholm2016.
15. Flink M, Ekstedt M. Planning for the Discharge, not for Patient Self-Management at Home - An Observational and Interview Study of Hospital Discharge. *Int J Integr Care.* 2017;17(6):1.
16. Gustafsson L, Bootle K. Client and carer experience of transition home from inpatient stroke rehabilitation. *Disability and rehabilitation.* 2013;35(16):1380-1386.
17. Lin MJ, Tirosh AG, Landry A. Examining patient comprehension of emergency department discharge instructions: Who says they understand when they do not? *Intern Emerg Med.* 2015;10(8):993-1002.

18. Ziaeian B, Araujo KL, Van Ness PH, Horwitz LI. Medication reconciliation accuracy and patient understanding of intended medication changes on hospital discharge. *Journal of general internal medicine.* 2012;27(11):1513-1520.
19. Riks-Stroke. *Stroke och TIA - ÅRSRAPPORT FRÅN RIKSSTROKE.* 2019.
20. Lorig KR, Holman H. Self-management education: history, definition, outcomes, and mechanisms. *Annals of behavioral medicine : a publication of the Society of Behavioral Medicine.* 2003;26(1):1-7.
21. Global, regional, and national burden of stroke, 1990-2016: a systematic analysis for the Global Burden of Disease Study 2016. *Lancet Neurol.* 2019;18(5):439-458.
22. World_Health_Organization. *Health Promotion Glossary.* Geneva1998.
23. Osborne RH, Batterham RW, Elsworth GR, Hawkins M, Buchbinder R. The grounded psychometric development and initial validation of the Health Literacy Questionnaire (HLQ). *BMC Public Health.* 2013;13:658.
24. Hoffmann T, McKenna K. Analysis of stroke patients' and carers' reading ability and the content and design of written materials: recommendations for improving written stroke information. *Patient Educ Couns.* 2006;60(3):286-293.
25. Berkman ND, Sheridan SL, Donahue KE, Halpern DJ, Crotty K. Low health literacy and health outcomes: an updated systematic review. *Annals of internal medicine.* 2011;155(2):97-107.
26. Aaby A, Friis K, Christensen B, Rowlands G, Maindal HT. Health literacy is associated with health behaviour and self-reported health: A large population-based study in individuals with cardiovascular disease. *Eur J Prev Cardiol.* 2017;24(17):1880-1888.
27. Bridgwood B, Lager KE, Mistri AK, Khunti K, Wilson AD, Modi P. Interventions for improving modifiable risk factor control in the secondary prevention of stroke. *Cochrane Database Syst Rev.* 2018;5:CD009103.
28. Goncalves-Bradley DC, Lannin NA, Clemson LM, Cameron ID, Shepperd S. Discharge planning from hospital. *Cochrane Database Syst Rev.* 2016(1):Cd000313.
29. Langhorne P, Baylan S. Early supported discharge services for people with acute stroke. *Cochrane Database Syst Rev.* 2017;7:Cd000443.
30. Leppin AL, Gionfriddo MR, Kessler M, et al. Preventing 30-Day Hospital Readmissions: A Systematic Review and Meta-analysis of Randomized Trials. *JAMA internal medicine.* 2014.
31. Ha Dinh TT, Bonner A, Clark R, Ramsbotham J, Hines S. The effectiveness of the teach-back method on adherence and self-management in health education for people with chronic disease: a systematic review. *JBI Database System Rev Implement Rep.* 2016;14(1):210-247.
32. Oh EG, Lee HJ, Yang YL, Kim YM. Effectiveness of Discharge Education With the Teach-Back Method on 30-Day Readmission: A Systematic Review. *J Patient Saf.* 2019.
33. Lindblom S, Flink M, Elf M, Laska AC, von Koch L, Ytterberg C. The manifestation of participation within a co-design process involving patients, significant others and health-care professionals. *Health Expect.* 2021.
34. Berthelsen O, Wiklund M, Säthil K, Samulowitz A, Fagevik Olsén M. An evaluation of two different methods for preoperative physical therapy information before abdominal surgery. *Journal of Communication in Healthcare.* 2020;13(2):102-110.
35. Craig P, Dieppe P, Macintyre S, Michie S, Nazareth I, Petticrew M. Developing and evaluating complex interventions: the new Medical Research Council guidance. *Bmj.* 2008;337:a1655.
36. Boyd H, McKernon S, Mullin B, Old A. Improving healthcare through the use of co-design. *N Z Med J.* 2012;125(1357):76-87.
37. Cohn S, Clinch M, Bunn C, Stronge P. Entangled complexity: why complex interventions are just not complicated enough. *J Health Serv Res Policy.* 2013;18(1):40-43.
38. Greenhalgh T, Papoutsi C. Studying complexity in health services research: desperately seeking an overdue paradigm shift. *BMC Med.* 2018;16(1):95.
39. Moore GF, Evans RE, Hawkins J, et al. From complex social interventions to interventions in complex social systems: Future directions and unresolved questions for intervention development and evaluation. *Evaluation (Lond).* 2019;25(1):23-45.

40. Elmstahl S, Malmberg B, Annerstedt L. Caregiver's burden of patients 3 years after stroke assessed by a novel caregiver burden scale. *Archives of physical medicine and rehabilitation.* 1996;77(2):177-182.
41. Elf M, Nordmark S, Lyhagen J, Lindberg I, Finch T, Aberg AC. The Swedish version of the Normalization Process Theory Measure S-NoMAD: translation, adaptation, and pilot testing. *Implementation science : IS.* 2018;13(1):146.
